# Lateral closed wedge osteotomy for cubitus varus deformity

**DOI:** 10.4103/0019-5413.43397

**Published:** 2008

**Authors:** Amit K Srivastava, DC Srivastava, SC Gaur

**Affiliations:** Department of Orthopaedics UCMS/GTB Hospital, Delhi, India; 1 MLN Medical College, Allahabad, India

**Keywords:** Cubitus varus deformity, fixation technique, lateral condylar wedge osteotomy

## Abstract

**Background:**

Lateral closed wedge (LCW) osteotomy is a commonly accepted method for the correction of the cubitus varus deformity. The fixation of osteotomy is required to prevent loss of correction achieved. The fixation of the osteotomy by the two screw and figure of eight wire is not stable enough to maintain the correction achieved during surgery. In this prospective study we supplemented the fixation by Kirschner's (K-) wires for stable fixation and evaluated the results.

**Materials and Methods:**

Twenty-one cases of the cubitus varus deformity following supracondylar fractures of the humerus were operated by LCW osteotomy during February 2001 to June 2006. The mean age of the patients at the time of corrective surgery was 8.5 years (range 6.6-14 years). The osteotomy was fixed by two screws with figure of eight tension band wire between them and the fixation was supplemented by passing two to three K-wires from the lateral condyle engaging the proximal medial cortex through the osteotomy site.

**Result:**

The mean follow-up period was 2.5 years (range seven months to 3.4 years). The results were assessed as per Morrey criteria. Eighteen cases showed excellent results and three cases showed good results. Two cases had superficial pin tract infection.

**Conclusion:**

The additional fixation by K wires controls rotational forces effectively besides angulation and translation forces and maintains the correction achieved peroperatively.

## INTRODUCTION

Cubitus varus is the most common delayed complication that results following supracondylar fracture of humerus in children. Immediate and late causes of cubitus varus deformity are medial angulations, medial rotation, overgro0wth of lateral condyle and osteonecrosis or delayed growth of medial condyle.^1^ The medial angulation is the major determinant for the deformity^2^ while medial rotation contributes to it.

There are several fixation techniques of corrective osteotomies of the distal humerus. The medial opening wedge osteotomy leads to instability and stretching of the ulnar nerve, and is difficult to fix.^3^ Lateral close wedge osteotomy (LCW) is the easiest, safest and inherently the most stable osteotomy. The dispute lies in the type of fixations which are most stable with minimum complications. The two screws and a figure of eight tension band wire attached between them, plate fixation, crossed Kirschner's (K-) wires, staples, external fixation and even no fixation are described in the literature. Roach *et al.*,^4^ reported the recurrence of deformity because of non-rigid fixation with K-wires and recommended the lateral two-hole plate and percutaneous medial pins to increase the stability. Oblique osteotomy fixed with cortical screws was also described by Amaspacher and Messenberg.^5^ Three-dimensional osteotomy was described by Uchida, Ogota and Sugioka.^6^ Voss *et al.*,^7^ did uniplanar supracondylar closing wedge osteotomy and fixed it with pre-set K-wire. French^8^ advocated lateral closing wedge osteotomy and fixation with two screws with figure of eight tension band wire between them. Bellemore^9^ modified French's technique by leaving the medial cortex short of the periosteum and fixing it like French's technique. Derosa and Gaziano^10^ reported good to excellent results after step-cut osteotomy and fixation with cortical screws. Sharma *et al.*,^25^ had performed pentalateral osteotomy; Rai^26^ used valgus rotation osteotomy; Levine *et al.*,^11^ Usai *et al.*,^12^ Handelsman *et al.*,^21^ Jain *et al.*^22^ and Goyal *et al.*,^23^ used unilateral external fixator to stabilize distal fragment after wedge osteotomy; Agarwal *et al.*,^24^ used biaxial external fixation; Song *et al.*^13^ and Karatosun *et al.*,^20^ used Ilizarov's technique with lateral closing wedge osteotomy in adults.

In the present prospective study we are reporting our experience in 21 cases treated by lateral closed wedge osteotomy and fixed by two screws and figure of eight tension band wire. The fixation was supplemented with two lateral K-wires.

## MATERIALS AND METHODS

Twenty-one cases of cubitus varus deformity following supracondylar fracture of the humerus, out of which 12 were male and nine were females, had been corrected by lateral close wedge osteotomy between February 2001 and June 2006. The osteotomy was first fixed by two screws and figure of eight tension band wire around them followed by supplementation of fixation with two to three K-wires passed through the lateral condyle up to the proximal medial cortex. The mean age at the time of injury was six years (range four to nine years) and at the time of corrective surgery was 8.5 years (range 6.6 to 14 years). Patients presented to us with loss of flexion by mean 11 degrees (range 0-20°) and two patients with hyperextension deformity of 14 and 16 degrees respectively. Clinically carrying angle was measured by angle formed between long axis of arm and forearm. The affected elbow was examined and compared with the contralateral side. Standard true anteroposterior and lateral radiograph of affected and normal elbow in identical position were used to assess the deformity and to do templating in every case. Radiologically, the humero-ulnar angle was taken into account. A varus angle of more than 10 degrees measured on radiograph and cosmetic complaints were considered as an indication for surgery. Preoperative templating has been done in each case. The cases in the relatively younger age group had been observed for a period of six months at monthly intervals, and had been assessed clinically and radiologically for any increase in deformity before contemplating surgery on them to exclude osteonecrosis or the overgrowth of any condyle. The cases were operated only when the deformity was considered static for six months in the follow-up before the age of 10 years. Postoperative radiographs were assessed at 1, 3, 6, 12 months time to asses the maintenance of correction achieved postoperatively.

The assessment of the outcome of the cases was done on the basis of Morrey^14^ criteria [Table 1].

**Table 1:** Morrey's system of functional assessment of outcome

	None	Mild	Moderate	Severe
Pain		If patient had occasional pain during use of the elbow but took no medication	If patient had pain at night occasionally took medication for pain but elbow did not limit the activity of daily living	If the patient took medication for pain regularly and activities of daily living were impaired
Stability		If varus valgus laxity was estimated to be less than 5 degrees and was not associated with any symptoms;	If varus valgus laxity was estimated to be less than 5-10 degrees and was associated with mild symptoms	If varus valgus laxity was estimated to be more than 10 degrees and was associated difficulty in activity of daily living
Motion	Flexion and extension of the elbow were measured with a hand goniometer held along the lateral aspect of the brachium and forearm. Pronation and supination were measured at the extremes of active motion, with one arm of the goniometer held along or parallel to the brachium and the second arm placed parallel to the dorsum or the volar aspect of the wrist		
Strength	Strength of flexion and extension was measured isometrically in all patients		

### Operative procedure

**Figure 1:**
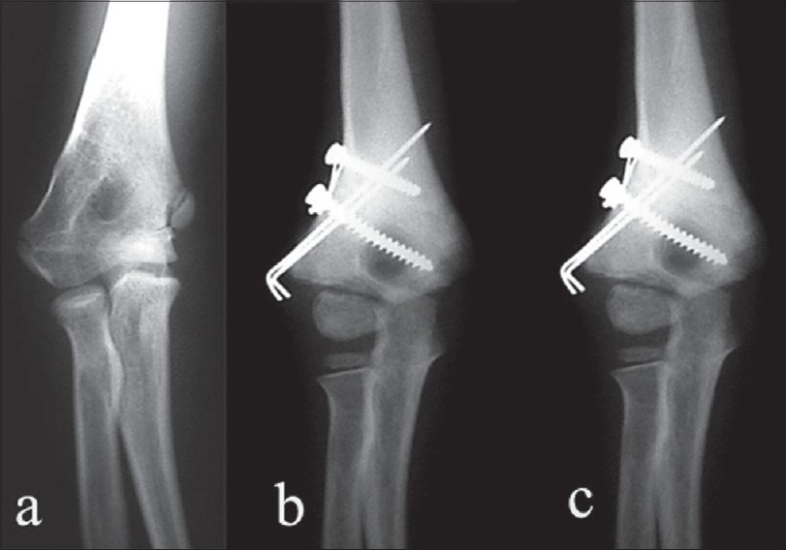
Radiograph (anteroposterior view) (a) Preoperative showing cubitus varus deformity. (b) Three months postoperative showing maintenance of correction and normal valgus achieved. (c) Eight months postoperative showing maintenance of correction.

After giving appropriate anesthesia, tourniquet was applied. The patient was positioned supine with the arm on a hand table. Posterolateral skin incision was made along the lower arm. The lateral third to half of the triceps muscle was reflected from its insertion. The osteotomy site was marked on the humerus with the help of the template which determines the length of the lateral wedge and angle of osteotomy. The desired correction was calculated by adding the differences of humero-ulnar angle of both elbows and adding normal valgus angle of normal elbow. The K-wire was inserted parallel to the proposed osteotomy site, one proximally and one distally. After checking the placement of K-wires under the C-arm, two cortical screws, one proximally and the other distally, were inserted parallel to the two K-wires. After removing measured wedge, the fragments were aligned with the help of pre-placed K-wires rather than aligning them by manipulating the forearm which usually does not provide the controlled force at the fracture site and may lead to break in the medial cortical hinge, and in turn to instability of fixation. The fixation had been secured with the help of figure of eight tension band wiring loop around the screws' heads after achievement of reduction of osteotomy and comparing it clinically with other elbow in full extension. This fixation was supplemented by two K-wires inserted from the lateral condyle passing through the osteotomy site and engaging the opposite proximal medial cortex [Figure 1]. The wound was closed and the above elbow plaster of paris slab was applied. Stitches were removed after 10 to 12 days of operation. Details about the 21 cases, including age, sex, follow-up period, rehabilitation period, carrying angle correction pre- and postoperatively are given in Table 2.

**Table 2:** The clinical details of patients

Case	Age (year)	Sex	Carrying angle (degree)		Carrying angle (degree)		Flexion (degree)		Duration of union (wks)	Carrying angle at the time of union (degree)	Results
								
			Pre op clinical varus angle	Pre op carrying angle of normal elbow	Immediate postop	At 12 week (followup)	Pre op	Post op (12 weeks)			
1	14	F	24	13	14	14	0-110	0-130	6.5	14	Excellent
2	10	M	25	11	12	12	5-115	0-120	8	12	Excellent
3	13	F	16	13	14	14	0-118	0-140	6	14	Excellent
4	8	F	18	15	14	14	0-125	0-138	5.5	14	Excellent
5	12	M	21	14	17	17	0-120	0-135	6	17	Excellent
6	11	M	22	13	15	15	5-145	0-145	7.5	15	Excellent
7	6	M	18	13	16	16	0-135	0-138	7	16	Good
8	9	F	24	14	14	14	0-120	0-130	8	14	Excellent
9	10	M	18	15	15	15	5-140	0-140	6	15	Excellent
10	6.5	M	20	11	15	15	10-126	0-130	6.5	15	Excellent
11	12.5	M	19	14	15	15	8-135	0-135	7.2	15	Excellent
12	12	F	21	12	13	13	0-121	0-129	6	13	Excellent
13	8	F	18	16	16	16	0-134	0-140	5.5	16	Good
14	9	M	19	12	14	14	0-120	0-130	7	14	Excellent
15	7.5	F	24	13	14	14	–5-120	0-138	8	14	Excellent
16	8.5	M	21	12	13	13	–14-105	0-132	6.5	13	Excellent
17	10	M	19	13	14	14	16-112	0-135	6	14	Excellent
18	12	M	18	12	16	16	0-125	0-140	7	16	Good
19	9	F	23	13	14	14	0-122	0-138	7.5	14	Excellent
20	8.5	M	23	14	12	12	0-118	0-131	6	12	Excellent
21	1.3	F	21	14	13	13	5-120	0-140	7	13	Excellent

Pre op - Pre operative, Post op - Post operative, M - Male, F - Female

## RESULT

The 21 cases were followed up to a mean period of 2.5 years (range seven months to 3.4 years). Twenty cases were fully satisfied with cosmetic results, but one case(no 10) had complaint related to cosmetic appearance. All the cases resumed their normal activity within three to six months of surgery. The radiological union at the osteotomy site took place in a mean period of 6.5 weeks (range 5.5 to 8 weeks). Preoperative mean loss of flexion of 11 degrees (range 0 to 20) reduced to mean of 3 degrees (range 0 to 5 degrees). The hyperextension in two cases by 14 and 16 degrees improved to normal postoperatively. The supination, pronation of forearm was same pre- and postoperatively. There was no pain in 16, mild pain in three, moderate pain in two cases and none had severe pain. A total of 20 patients (95%) were satisfied with the cosmetic appearance while one (case no. 10) (5%) noticed little difference due to excessive lateral condylar prominence. Of the 21 patients, 19 were able to have full range of motion after a mean of 6.8 weeks (range 5.5 to 8.4 weeks), while two patients regained at 9 and 10 weeks respectively. No case had instability in the coronal plane.

Eighteen (85%) patients showed excellent results, three (15%) good while none showed fair or poor results in the follow-up. Statistical analysis was not done due to the small study group. None of our patients had any neurovascular deficit postoperatively. There was superficial pin tract infection in two cases (case no.7 and 13) but it responded to local wound care and antibiotics. None reported pin loosening, gross loss of fixation, and loss of correction. The comparison of the results of various other methods with our method is given in Table 3)

**Table 3:** Comparative table for complications produced in different types of fixations recommended by different authors

Studies	Fixation type	Complication
King and Secor^3^ (15 cases)	MOW, Reidel clamp and graft	Ulnar palsies (n=3), aneurysm (n=1), Cubitus rectus (n=3), skin slough (n=1)
Rang^16^ (20 cases)	LCW, K-wires	Varus (6), stiffness (2)
McCoy and Piggot^17^ (20 cases)	LCW, French method	4-neutral, 2-varus, 2-stiffness
Graham *et al*. (16 cases)	LCW and cast	Varus (n=2)
Oppenheim^18^*et al*. (45 cases)	LCW and K-wire with screw	Nerve palsies (n=5), infections (n=3), varus (n=12)
Derosaand Graziano^10^ (11 cases)	Step cut and screw	Loss of fixation (n=1)
Kannujia^19^*et al*. (11 cases)	Dome osteotomy and K- wire	Stiffness (n=2)
Uchida *et al*. (12 cases)	Steop cut and screw	None
Voss and Kasser (34 cases)	K-wires	Loss of fixation (n=1)
Present study (21 cases)	LCW, screws and steel wires, 2 K- wires	None (only superficial pin tract infection in 2 cases)

MOW - Medial Opening Wedge, LCW - Lateral Closing Wedge

**Figure 2:**
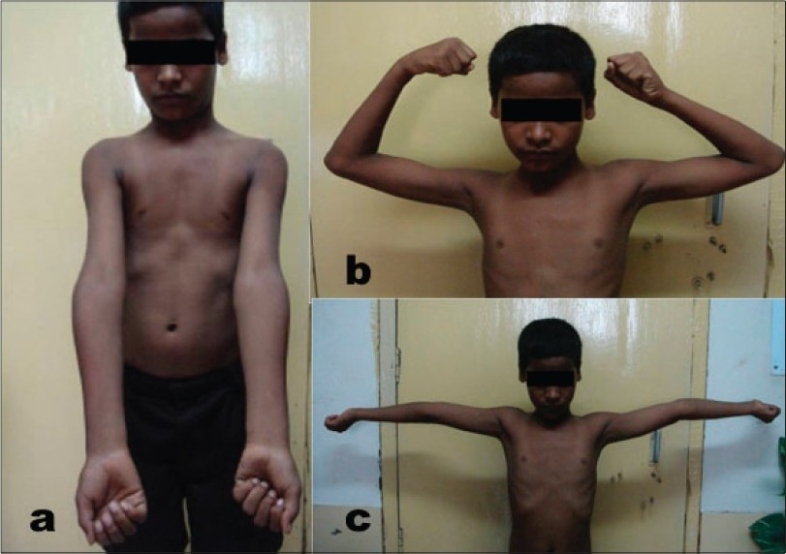
(a) Clinical photograph showing good correction of right elbow deformity. (b, c) Clinical photograph showing good range of movement.

Average preoperative varus was 20.1 degrees (range 16-25), immediate postoperative and 12^th^ week postoperative valgus angle measured 14.4 degrees (range 12-17 degrees). The radiological valgus achieved on the operated side was near equal to valgus of normal side with a mean variation of ±1.91 degrees (range - 2 in case no. 20 to + 4 degrees in case no. 10 at 12-week follow-up [Table 2]). Cosmetically all were satisfied with the outcome. There had been no neurovascular complication, unsightly scar or any residual deformity. Stable fixation had led our most of the cases to achieve >170 degree of supination- pronation, <5-10 degrees of restriction of flexion-extension in the majority of the cases [Figure 2]. Most of our patients were able to regain their pre-injury functional status in the ninth week postoperatively with excellent cosmetic correction.

## DISCUSSION

Lateral close wedge (LCW) osteotomy is the easiest, safest and inherently stable method of correction. The type of fixation of osteotomy is a concern to achieve good result. Roach *et al.*,^4^ believed that unstable, non-rigid fixation led to slip of the fragments and loss of correction. Various methods of fixation are: use of two screws and figure of eigth tension band wire attached to them, plate fixation, cross K-wire fixation, staples; few authors used no fixation. The fixation by crossed K-wires frequently led to loosening of the fixation with recurrence of deformity,^10^ pin tract infection^18^ skin slough,^3^ nerve palsy^3^,^18^ and rarely brachial artery aneurysm.

This modification reported by us to stabilize lateral closed wedge osteotomy for the cubitus varus deformity has certain advantages. We have used two K-wires in addition to two screws with TBW at the osteotomy site which gives us more control on the proximal and distal fragments which avoids the fracture of the medial cortex after closing the osteotomy. In addition, on peroperative clinical evaluation it gives better control of translation, rotation and angulations. Thus this method of fixation reduces the chance of the recurrence of the deformity. We respected the periosteum by not stripping it too much, thus giving the osteotomy more biological environment for fast healing. Experience has shown that the biological determinants of fracture healing are as important as the mechanical and must be respected.^15^

The K-wires were passed across the osteotomy site when the elbow was in position of full extension thus ensuring that there was no mechanical block postoperatively for regaining full elbow movements. In two cases with hyperextension deformity, the K-wires were passed when the elbow was at zero degree of extension; an appropriate anterior wedge was also removed before the fixation of osteotomy.

Roach *et al.*,^4^ recommended two-hole plates on the lateral site and two K-wires from the medial side for providing stable fixation but this produced more nerve palsies and two-hole plate does not give the strong axial hold to the proximal and distal fragments. We had no nerve palsies in our study. According to Sang *et al.*, the maintenance of early movement during treatment in order to obtain good functional results is the most important consideration. This problem is encountered when prolonged cast immobilization is required if the fixation is bio-mechanically not stable. In our study the addition of two K-wires, residual medial cortical hinge and the two screws with wires around them had made the fixation more stable and no loss of correction occurred. None had reported poor cosmetic result till the recent follow-up except one case (no-10) due to lateral condylar prominence.

We believe that the modified method of fixation is a simple reliable, acceptable and effective method. In our series using this fixation method, peroperatively achieved correction is not lost till the union is achieved.
